# Supplementary material for: Developing and validating a self-assessment tool for assessing confidence of nurse-midwives against competency domains of the International Confederation of Midwives, in India
Source: PLOS Glob Public Health. 2024 Oct 23;4(10):e0003766. doi: 10.1371/journal.pgph.0003766 (PMC11498683; doi:10.1371/journal.pgph.0003766)
Supplement: S1 File — (PDF) [file pgph.0003766.s002.pdf]

Competency assessments for midwifery skills of providers and tutors, and barriers/facilitators in providing quality midwifery services in India

BACKGROUND INFORMATION OF PRACTITIONERS

<table>
<tr><th colspan="3"></th><th colspan="2">NAME</th><th>Survey CODE</th></tr>
<tr><td colspan="3">STATE</td><td colspan="2"></td><td></td></tr>
<tr><td colspan="3">DISTRICT</td><td colspan="2"></td><td></td></tr>
<tr><td colspan="3">Institute Name</td><td colspan="2"></td><td></td></tr>
<tr><td colspan="3">Type of Institute</td><td colspan="3"></td></tr>
<tr><td colspan="5">Respondent ID number</td><td></td></tr>
<tr><td>INTERVIEW DATE</td><td colspan="2">DAY
[ ][ ]</td><td>MONTH
[ ][ ]</td><td colspan="2">YEAR
[ ][ ][ ][ ]</td></tr>
<tr><td>NAME OF INVESTIGATOR</td><td colspan="5"></td></tr>
<tr><td>CODE OF INVESTIGATOR</td><td colspan="5">[ ][ ][ ]</td></tr>
<tr><td>SIGNATURE OF INVESTIGATOR</td><td colspan="5"></td></tr>
</table>

Competency assessments for midwifery skills of providers and tutors, and barriers/facilitators in providing quality midwifery services in India

BACKGROUND INFORMATION SHEET FOR PRACTITIONERS

**Name of the researcher:**		**Date:**	
**No.**	**Details**	**Options**	
1	Age (in years)		
2	Sex	Male---------------------1	
		Female--------------------2	
		Any other please specify..3	
3	Please mention your highest qualification	BSc nursing…………..1	
		General Nursing and midwifery…………….2	
		Post basic BSc………...3	
		Master's programme……4	Mention specialization
		Nurse Practitioners in midwifery………………5	
4	Please list the various training programmes attended by you in the last 3 years		
5	How many months/years of total clinical experience do you have?	No experience…….0<br>Yes…..1 Months:	Years:
6	How months/years of clinical experience do you have in midwifery?	No experience…….0<br>Yes…..1 Months:	Years:
7	Roughly how many childbirths do you attend per week?		
