# Supplementary material for: Developing and validating a self-assessment tool for assessing confidence of nurse-midwives against competency domains of the International Confederation of Midwives, in India
Source: PLOS Glob Public Health. 2024 Oct 23;4(10):e0003766. doi: 10.1371/journal.pgph.0003766 (PMC11498683; doi:10.1371/journal.pgph.0003766)
Supplement: S2 File — (PDF) [file pgph.0003766.s003.pdf]

SELF-ASSESSMENT OF MIDWIFERY COMPETENCIES OF MIDWIFERY PRACTITIONERS (GNM/ B.Sc./M.Sc.)

Competency assessments for midwifery skills of providers and tutors, and barriers/facilitators in providing quality midwifery services in India.

Please read the description given below carefully before responding to the questionnaire

In many parts of world midwives are the first contact for women when they conceive. Midwives have an independent practice with full autonomy of handling normal childbirth. The International Confederation of Midwives (ICM) and WHO have given a standard list of competencies for midwives to provide good quality maternity care to women and newborns. The Government of India is committed to establish childbirth services which are led by midwives. They have released national guidelines for midwife-led care. Staff nurses who are interested to upgrade themselves as dedicated midwives will have to undergo an 18 months training. These trained midwives will be in-charge of normal childbirth in health facilities; conduct Antenatal OPD, assist normal birth independently, provide postnatal care and will have the right to admit, prescribe laboratory tests and discharge women on their own responsibility.

For this reason the Government of India and WHO are keen to assess current levels of midwifery competencies of staff nurses with GNM diploma, B.Sc.(N) and/or M.Sc.(N), practicing in the maternity sections. Through this questionnaire we ask your help in identifying competencies for which staff nurses have confidence and competencies where there is a need for further education. This study is being undertaken in six states of India.

About the questionnaire: This questionnaire has been adapted from the list of essential midwifery competencies given by ICM. There are seven domains of competencies with many skills and behaviours under each, given as separate tables. In addition questions have been included to capture characteristic features of current policy/situation/practice.

For each competency, there is a slider bar given where you can mark your SELF ASSESSMENT IN GENERAL about your existing knowledge of underlying theories, concepts, models of practice, and published evidence related to midwifery.

Please note that you donot need to give your name or any other identifying information. We once again assure you that your participation in the study is voluntary. You have the freedom to decline participation at any point. This is not an evaluation of either you or your institution. Your responses will help us in accurate gap analysis for midwifery competence which will help in making specific recommendations for measures the government needs to take to improve midwifery education and practice. Findings from this research will be collective, so individual responses will remain confidential.

Please help us by filling out this self-assessment as accurately and honestly as you can. A member of our research team is around you while you respond to this questionnaire. Please do not hesitate to ask them for clarification in case of any doubt. Thank you so much for your time.

1. **Midwives have the requisite knowledge and skills from obstetrics, neonatology, the social sciences, public health and ethics that form the basis of high quality, culturally relevant, appropriate care for women, newborns, and childbearing families.**

Based on my own assessment of my knowledge, **in general** I rate my knowledge of obstetrics, neonatology, the social sciences, public health and ethics needed to provide high quality, culturally-relevant and appropriate care for women-newborns and childbearing families as:

(Move the slider to rate your self-assessment of knowledge in this area on a scale of 0 to 100 OR place a cross on the line below to mark your assessment of self-knowledge on a scale of 0-100 WHERE the left end point of line denotes 0 knowledge and the right endpoint of the line denotes 100% knowledge)

**Item No. A**	**Skill Statements:**<br><br>**Midwives have the requisite knowledge and skills from obstetrics, neonatology, the social sciences, public health and ethics that form the basis of high quality, culturally relevant, appropriate care for women, newborns, and childbearing families**	**Not aware of this skill/not relevant**<br><br>**(Please tick√)**	**How confident are you to perform this skill independently?**<br>**1: Not confident**<br>**2: Little confident**<br>**3: Somewhat confident**<br>**4: Quite confident**<br>**5: Very Confident**<br>**(Please mark your self-rating)**
A01	I can accurately assess the social determinants of health and illness in my community		
A02	I can accurately assess the community determinants of health and illness in my community		
A03	I can identify gaps in my ward/labour room for quality care		
A04	I can engage with appropriate authorities to ensure my clinical area is well prepared		
A05	I can engage women and families in health promotion activities/discussions		
A06	I can effectively control/prevent infection in my clinical area		
A07	I take responsibility for making clinical decisions for women/newborn babies		
A08	I can identify cultural practices important for women (religious beliefs, rituals etc.)		
A09	I can identify the social reasons behind why some women can take independent decisions and some not		
A10	I can engage in health education discussions with and for women and their families		
A11	I can use appropriate communication and listening skills when interacting with women		
A12	I can assemble the instruments needed to care for women and newborn babies (eg: Baby tray, delivery tray, Ambu bag etc.)		
A13	I can use the instruments needed to care for women and newborn babies		
A14	I can maintain and care for the instruments used in obstetric and newborn care (eg. Proper cleaning, storage, repair etc)		

Item No. A	Skill Statements:<br><br>Midwives have the requisite knowledge and skills from obstetrics, neonatology, the social sciences, public health and ethics that form the basis of high quality, culturally relevant, appropriate care for women, newborns, and childbearing families	Not aware of this skill/not relevant<br><br>(Please tick√)	How confident are you to perform this skill independently?<br>1: Not confident<br>2: Little confident<br>3: Somewhat confident<br>4: Quite confident<br>5: Very Confident<br>(Please mark your self-rating)
A15	I can record my clinical observations in medical documents		
A16	I can report the services provided based on my clinical observations		
A17	I can interpret the medical reports		
A18	I can plan the follow-up required by women/newborn babies based on medical reports		
A19	I comply with all local reporting regulations for birth and death registration in India		
A20	I know what procedures/services are legally allowed for midwifery practice and can practice within the legal framework in India		
	**I can identify cases of:**		
A21	- Domestic violence		
A22	- Rape		
A23	- Genital mutilation; male/female (Cutting off clitoris of girls)		
A24	I can refer the victims of rape/domestic violence/genital mutilation to appropriate agencies for further services		
A25	I can effectively campaign/advocate for normal labor & birth		
A26	I can empower women to ask for normal physiologic labor & birth		

2. **Midwives provide high quality, culturally sensitive health education and services to all in the community in order to promote healthy family life, planned pregnancies and positive parenting**

a. Are you allowed to prescribe family planning method independently to couples in your care **Yes** ☐ **No** ☐

b. If yes, which methods can you independently prescribe? (Tick all that apply):

Barrier method ☐ **Steroidal method** ☐ **Mechanical method** ☐ **Chemical method** ☐ **Emergency contraception** ☐

c. **Based on my own assessment of my knowledge, in general I rate my knowledge regarding theories and principles for providing high quality, culturally sensitive health education and services to all in the community and to promote healthy family life, planned pregnancies and positive parenting as:**

(Move the slider to rate your self-assessment of knowledge in this area on a scale of 0 to 100 OR place a cross on the line below to mark your assessment of self-knowledge on a scale of 0-100 WHERE the left end point of line denotes 0 knowledge and the right endpoint of the line denotes 100% knowledge)

**Item No. B**	**Skill Statements**<br><br>**Midwives provide high quality, culturally sensitive health education and services to all in the community in order to promote healthy family life, planned pregnancies and positive parenting**	**Not aware of this skill/not relevant**<br><br>**(Please tick√)**	**How confident are you to perform this skill independently?**<br>**1: Not confident**<br>**2: Little confident**<br>**3: Somewhat confident**<br>**4: Quite confident**<br>**5: Very Confident**<br>**(Please mark your self-rating)**
	**GENERAL EXAMINATION**		
B01	I can collect a comprehensive reproductive health history		
	**I can collect history about following areas:**		
B02	- Sexual growth and development (eg: sexual activity with partner)		
B03	- Male reproductive anatomy and physiology (eg: Infertility)		
B04	- Female reproductive anatomy and physiology		
B05	- Menstrual history		
B06	I can perform a complete general physical examination of pregnant women		
B07	I can identify deviations from normal anatomy/ physiology		
B08	I can perform commonly required laboratory tests /order relevant laboratory tests based on physical examination findings (example: urine test, hematocrit/ESR, and others)		
B09	I can accurately interpret the findings of commonly required laboratory tests		
B10	I can identify key signs of specific disease during a physical examination (eg: Anaemia, Jaundice, High blood pressure etc.)		
B11	I can accurately interpret the findings of specific tests required		
B12	I can take accurate preventive measures in case of exposure to HIV for myself and co-workers		
	**FAMILY PLANNING**		
	**I can carry out an appropriate counselling for the couples on following topics:**		
B13	- Pre-natal Counselling		

B14	- Counselling on male family planning methods		
B15	- Counselling for female family planning methods		
B16	- HIV test-related counselling		
B17	- Counselling to HIV positive couple/man/woman		
B18	I can identify from the couples responses the cultural/social issues that will influence their decision-making processes		
B19	I can make my counselling sessions culturally sensitive		
B20	I provide comprehensive advice to the couple on side effects associated with different family planning methods		
B21	I can prescribe the family planning method selected by the couple		
B22	I can insert an intrauterine device		
B23	I can remove an intrauterine device		
B24	I can insert a contraceptive implant		
B25	I can remove a contraceptive implant		
B26	I can perform cervical cytology test (PAP smear)		
B27	I can perform HPV test		
B28	I can use the microscope to perform simple screening tests		
B29	I can perform acetic acid visualization of cervix		
B30	I can interpret findings from acetic acid test		
B31	I can perform a colposcopy test for cervical cancer screening		
B32	I can interpret findings from colposcopy test		

3. **Midwives provide high quality antenatal care to maximize health during pregnancy and that includes early detection and treatment or referral of selected complications.**

a. The regulations guiding midwifery care presently allow me to prescribe drugs ☐ Yes ☐ No

b. If yes, I can prescribe following drugs (indicate by placing a tick next to the drug group):

Antibiotic ☐ Anticonvulsant ☐ Antimalarial ☐ Antihypertensive ☐ Antiretroviral ☐

Any other (please specify here) ____

c. Based on my own assessment of my knowledge, **in general** I rate **my knowledge** needed for providing high quality **antenatal care to maximize health during pregnancy, including early detection and treatment or referral of selected complications** as:

(Move the slider to rate your self-assessment of knowledge in this area on a scale of 0 to 100 OR place a cross on the line below to mark your assessment of self-knowledge on a scale of 0-100 WHERE the left end point of line denotes 0 knowledge and the right endpoint of the line denotes 100% knowledge)

**Item No. C**	**Skill Statements:**<br><br>**Midwives provide high quality antenatal care to maximize health during pregnancy and that includes early detection and treatment or referral of selected complications**	**Not aware of this skill/not relevant**<br><br>**(Please tick√)**	**How confident are you to perform this skill independently?**<br>**1: Not confident**<br>**2: Little confident**<br>**3: Somewhat confident**<br>**4: Quite confident**<br>**5: Very Confident**<br>**(Please mark your self-rating)**
C01	I can accurately identify signs and symptoms of pregnancy		
C02	I can accurately collect the antenatal history of the woman in my care		
C03	I can accurately perform test to confirm pregnancy (urine pregnancy test)		
C04	I can accurately calculate the estimated date of birth (using LMP, and/or USG findings)		
C05	I can diagnose an ectopic pregnancy		
C06	I can accurately measure maternal vital signs (pulse, respiration, temperature, blood pressure)		
	**I can accurately identify following parameters to assess maternal nutrition:**		
C07	- Pallor		
C08	- Capillary filling time		
C09	- Weight and weight-gain		
C10	- Signs of nutritional deficiency		
C11	- Signs of substance abuse (alcohol, smoking, other)		
C12	I can advise the woman on correct nutritional intake including iron-folic-acid, calcium, zinc supplementation		
C13	I can successfully give de-worming treatment (administer medicine for worms) to women during pregnancy		
C14	I can help the woman in developing a culturally sensitive and affordable diet plan		
	**I can perform an accurate abdominal examination including:**		
C15	- Fundal height		
C16	- Abdominal girth		
C17	- Fetal position		

Item No. C	**Skill Statements:**<br><br>**Midwives provide high quality antenatal care to maximize health during pregnancy and that includes early detection and treatment or referral of selected complications**	**Not aware of this skill/not relevant**<br><br>**(Please tick√)**	**How confident are you to perform this skill independently?**<br>**1: Not confident**<br>**2: Little confident**<br>**3: Somewhat confident**<br>**4: Quite confident**<br>**5: Very Confident**<br>**(Please mark your self-rating)**
C18	- Fetal lie		
C19	- Assessment of pelvic adequacy		
C20	- Multiple gestation		
C21	- Oligo/polyhydramnios		
C22	- Intrauterine growth restrictions		
C23	- Locating and auscultating Fetal heart rate		
C24	Identification of signs of complications		
C25	I can accurately use the Doppler to check the fetal heart sounds		
C26	I can accurately identify deviation from normal for ALL the above-mentioned parameters		
C27	I can independently perform an ultrasound examination		
C28	I can identify/evaluate the parameters from an ultrasound examination		
C29	I can accurately perform pelvic assessment for cephalo-pelvic disproportion		
	**I can counsel the woman and her family on**		
C30	- Progression of normal pregnancy		
C31	- Key signs to note and report to care providers		
C32	- Identify Braxton Hick contractions		
C33	- Breast changes during pregnancy		
C34	- Assessment of daily fetal movement		
C35	- Identify the danger signs during pregnancy		
C36	- How and when to contact a health care provider		
C37	- Common ailments during pregnancy		
C38	- Managing common ailments of pregnancy at home		
C39	- Basic preparation for labour (breathing exercise, positions, non-pharmacologic comfort measures etc.)		
C40	- Birth preparedness and complication readiness		
C41	- Basic parenting skills		
C42	- Breastfeeding		

Item No. C	Skill Statements:<br><br>Midwives provide high quality antenatal care to maximize health during pregnancy and that includes early detection and treatment or referral of selected complications	Not aware of this skill/not relevant<br><br>(Please tick√)	How confident are you to perform this skill independently?<br>1: Not confident<br>2: Little confident<br>3: Somewhat confident<br>4: Quite confident<br>5: Very Confident<br>(Please mark your self-rating)
	**I can identify following complications the woman for:**		
C43	- Hemorrhage during pregnancy (abortion)		
C44	- Hemorrhage during late pregnancy (placenta previa)		
C45	- Pre-Eclampsia		
C46	- Eclampsia		
C47	- Gestational Diabetes		
C48	- Malaria		
C49	- Psychological stress		
C50	- Antenatal depression		
C51	- Fear of childbirth		

4. **Midwives provide high quality, culturally sensitive care during labour, conduct a clean and safe birth and handle selected emergency situations to maximize the health of women and their newborns**.

a. I wait for ____ hours between two per vaginal examinations   b. I delay the cord clamping for ____ minutes

b. Based on my own assessment of my knowledge, **in general** I rate **my knowledge** of theories and principles that support provision of **high quality, culturally sensitive care during labour, conduct a clean and safe birth and handle selected emergency situations to maximize the health of women and their newborns** to be as:

(Move the slider to rate your self-assessment of knowledge in this area on a scale of 0 to 100 OR place a cross on the line below to mark your assessment of self-knowledge on a scale of 0-100 WHERE the left end point of line denotes 0 knowledge and the right endpoint of the line denotes 100% knowledge)

Item No. D	Skill Statements<br><br>**Midwives provide high quality, culturally sensitive care during labour, conduct a clean and safe birth and handle selected emergency situations to maximize the health of women and their newborns**	Not aware of this skill/not relevant<br><br>(Please tick√)	How confident are you to perform this skill independently?<br>1: Not confident<br>2: Little confident<br>3: Somewhat confident<br>4: Quite confident<br>5: Very Confident<br>(Please mark your self-rating)
D01	I can follow infection control procedures based on national/local guidelines during Intrapartum care (eg: Protocols related to washing, waste disposal, sterilization of surfaces and equipment)		
D02	I can take specific history & maternal vital signs in labour (BP, Temp, Pulse, Resp)		
D03	I can perform focused physical examination during labour		
D04	I can calculate time of uterine contractions		
D05	I can assess effectiveness of uterine contractions		
D06	I can perform complete/accurate pelvic examination for dilatation, descent, presenting part, position, membranes status, & pelvis for vaginal birth		
D07	I can monitor progress of labour using partograph or similar tool for recording		
D08	I can provide physical & psychological support for woman and family & promote normal birth		
D09	I can help the presence of birth companion during labour and birth		
D10	I can ensure adequate hydration, nutrition & non-pharmacological comfort measures in labour/birth		
D11	I can provide for bladder care including performance of urinary catheterization when indicated		
D12	I can identify abnormal (eg. prolonged) labour patterns and initiate appropriate and timely intervention and/or referral		
D13	I can stimulate or augment uterine contractility, without drugs such as oxytocin		
D14	I can prepare for birth (woman and partner/family, equipments, labour room, etc.)		
D15	I can administer local anaesthetic to perineum for episiotomy or perineal repair.		
D16	I can perform an episiotomy		
D17	I can perform appropriate hand manoeuvres for a head birth		
D18	I can perform appropriate hand manoeuvres for face and breech deliveries		
D19	I can clamp and cut the cord correctly		
D20	I can manage prolapsed cord till the doctors come and/or waiting for transfer		
D21	I can manage mal-presentation, while requesting medical attention and/or awaiting transfer		

Item No. D	Skill Statements<br><br>**Midwives provide high quality, culturally sensitive care during labour, conduct a clean and safe birth and handle selected emergency situations to maximize the health of women and their newborns**	Not aware of this skill/not relevant<br><br>(Please tick√)	How confident are you to perform this skill independently?<br>1: Not confident<br>2: Little confident<br>3: Somewhat confident<br>4: Quite confident<br>5: Very Confident<br>(Please mark your self-rating)
D22	I can manage shoulder dystocia, while requesting medical attention and/or awaiting transfer		
D23	I can manage foetal distress, while requesting medical attention and/or awaiting transfer		
D24	I can manage cord around baby's neck at birth		
D25	I can conduct active management of the 3rd stage of labour; Administer uterotonic drug within a minute of birth of infant		
D26	I can inspect placenta and membranes for completeness		
D27	I can perform fundal massage to stimulate postpartum uterine contraction & uterine tone.		
D28	I can provide a safe environment for mother and infant to promote attachment (bonding)		
D29	I can estimate & record maternal blood loss		
D30	I can inspect the vagina and cervix for lacerations		
D31	I can repair 1st and 2nd degree vaginal tears or episiotomy if needed		
D32	I can manage postpartum bleeding & hemorrhage, using appropriate techniques & uterotonic agents		
D33	I can administer prescribed drugs or drugs as per the national protocols and guidelines		
D34	I can perform manual removal of placenta		
D35	I can perform aortic compression		
D36	I can identify signs of shock		
	**Initiate management of shock**		
D37	- Intravenous,		
D38	- Oxygen,		
D39	- Warmth,		
D40	- Position		-
D41	I can insert intravenous line	-	-
D42	I can draw blood for laboratory testing		
D43	I can arrange for timely referral/ transfer of women with serious complications to higher level health facility		
D44	I can perform adult cardio-pulmonary resuscitation		
D45	I can identify cervical lacerations and provide first level care before referral		

Item No.<br>D	Skill Statements<br><br>**Midwives provide high quality, culturally sensitive care during labour, conduct a clean and safe birth and handle selected emergency situations to maximize the health of women and their newborns**	Not aware of this skill/not relevant<br><br>(Please tick√)	How confident are you to perform this skill independently?<br>1: Not confident<br>2: Little confident<br>3: Somewhat confident<br>4: Quite confident<br>5: Very Confident<br>(Please mark your self-rating)
D46	I can provide HIV regime to HIV positive women as per national guidelines		
D47	I can document diagnosis and care in appropriate registers		
	**I can provide respectful maternity care to EVERY woman during labour and childbirth:**		
D48	- I respect the woman's integrity		
D49	- I respect her privacy at all times		
D50	- I seek permission before I touch the woman		
D51	- I regularly inform the woman about progress of labour		
D52	- I seek woman's input in planning her care		
D53	- I respect the newborn baby's integrity and privacy		
D54	- I avoid unnecessary discomfort to woman during any procedure		
D55	- I can provide opportunity for women to express their needs, choices during labour		

5. **Midwives provide comprehensive, high quality, culturally sensitive postpartum care for women**

a. Based on my own assessment of my knowledge, **in general** I rate **my knowledge** of theories and principles for provision of **comprehensive, high quality, culturally sensitive postpartum care for women** to be as:

(Move the slider to rate your self-assessment of knowledge in this area on a scale of 0 to 100 OR place a cross on the line below to mark your assessment of self-knowledge on a scale of 0-100 WHERE the left end point of line denotes 0 knowledge and the right endpoint of the line denotes 100% knowledge)

Item No.E	Skill Statements<br><br>**Midwives provide comprehensive, high quality, culturally sensitive postpartum care for women**	Not aware of this skill/not relevant<br><br>(Please tick√)	How confident are you to perform this skill independently?<br>1: Not confident<br>2: Little confident<br>3: Somewhat confident<br>4: Quite confident<br>5: Very Confident<br>(Please mark your self-rating)
E01	I can perform the comprehensive postpartum physical examination		
	**I can screen women for following postpartum complications such as:**		
E02	- Postpartum shock		
E03	- Hemorrhage		
E04	- Sepsis		
E05	- Haematoma formation		
E06	- Postnatal eclampsia		
E07	- Urinary retention		
E08	- Incontinence of feces or urine		
E09	- Obstetric fistula		
E10	- Episiotomy wound gaping		
E11	I can screen all postnatal women for signs of depression		
	**ADVICES GIVEN TO WOMEN**		
	**I can counsel postnatal women/couple/family on:**		
E12	- Emotional support required in postnatal period		
E13	- Physical self-care (including hygiene, safer sex)		
E14	- Physical neonatal care (hygiene, maintaining body temperature etc.)		
E15	- Warning signs and symptoms to be reported to care providers		
E16	- Physiological changes expected in postnatal period		
E17	- Regaining perineal muscle strength		
E18	- Women's nutritional needs in postnatal period (including iron-folic acid supplementation)		
E19	I can counsel for lactation and breastfeeding		
E20	I can initiate and support effective breastfeeding within the first hour of birth		
E21	I can assess the signs of breast engorgement, nipple sores/cracked nipples etc.		
E22	I can effectively aid the woman to manage breast related complications (compresses, breast pumps, nipple cover etc.)		

Item No.E	Skill Statements<br><br>**Midwives provide comprehensive, high quality, culturally sensitive postpartum care for women**	**Not aware of this skill/not relevant**<br><br>**(Please tick√)**	**How confident are you to perform this skill independently?**<br>**1: Not confident**<br>**2: Little confident**<br>**3: Somewhat confident**<br>**4: Quite confident**<br>**5: Very Confident**<br>**(Please mark your self-rating)**
E23	I can effectively teach the women how to express and store breast milk		
E24	I can counsel the couple grieving after loss of a newborn		
E25	I can counsel a family after a maternal loss		
	**I can accurately assess for the:**		
E26	- involution of uterus		
E27	- nature of lochia		
E28	- nature of postpartum bleeding		
E29	- signs of postnatal infection		
E30	- signs of thrombophlebitis		
E31	I can provide first line of treatment for postpartum complications		
E32	I can provide emergency care to/ stabilize and refer a woman with postpartum complication		
E33	I can perform manual vacuum aspiration for late-onset postpartum haemorrhage		

6. **Midwives provide high quality, comprehensive care for the essentially healthy infant from birth to two months of age.**

a. Based on my own assessment of my knowledge, **in general** I rate **my knowledge** of theories and principles that support provision of **high quality, comprehensive care for the essentially healthy infant from birth to two months of age** to be as:

(Move the slider to rate your self-assessment of knowledge in this area on a scale of 0 to 100 OR place a cross on the line below to mark your assessment of self-knowledge on a scale of 0-100 WHERE the left end point of line denotes 0 knowledge and the right endpoint of the line denotes 100% knowledge)

Item No.F	Skill Statements<br><br>**Midwives provide high quality, comprehensive care for the essentially healthy infant from birth to two months of age**	Not aware of this skill/not relevant<br><br>(Please tick√)	How confident are you to perform this skill independently?<br>1: Not confident<br>2: Little confident<br>3: Somewhat confident<br>4: Quite confident<br>5: Very Confident<br>(Please mark your self-rating)
F01	I can follow infection control procedures based on national/local guidelines during newborn care (e.g. Protocols related to washing, waste disposal, sterilization of surfaces and equipment)		
F02	I can provide immediate care to the newborn, including cord clamping and cutting, drying, clearing airways, and ensuring that breathing is established		
F03	I can assess immediate condition of newborn (e.g., APGAR scoring or other assessment method of breathing and heart rate)		
F04	I can promote & maintain normal newborn body temperature through covering (blanket, cap), environmental control, & promotion of skin-to-skin contact		
F05	I can begin emergency measures for respiratory distress (newborn resuscitation)		
F06	I can begin emergency measures for hypothermia		
F07	I can begin emergency measures for hypoglycaemia		
F08	I can give appropriate care to the low birth weight baby including kangaroo mother care		
F09	I can identify complications of low birth weight and refer		
F10	I can perform a screening/physical examination of newborn for congenital defects		
F11	I can provide routine newborn care, as per local guidelines and protocols (e.g., identification, eye care, screening tests, administration of vitamin k, birth registration)		
F12	I can position infant to initiate breast feeding as soon as possible after birth and support exclusive breastfeeding		
F13	I can transfer the at-risk newborn to appropriate emergency care facility		
F14	I can educate parents about normal growth and development of the infant and young child, and how to provide for day-to-day needs of the normal child		
F15	I can assist parents to access community resources available to the family (Eg. Anganwadi, Subcentre etc)		

Item No.F	Skill Statements<br><br>**Midwives provide high quality, comprehensive care for the essentially healthy infant from birth to two months of age**	**Not aware of this skill/not relevant**<br><br>**(Please tick√)**	**How confident are you to perform this skill independently?**<br>**1: Not confident**<br>**2: Little confident**<br>**3: Somewhat confident**<br>**4: Quite confident**<br>**5: Very Confident**<br>**(Please mark your self-rating)**
F16	I can support parents during transport/transfer of newborn or during times of separation (e.g. NICU admission)		
F17	I can support and educate parents who have given birth to multiple babies (e.g., twins, triplets) about special needs and community resources		
F18	I can provide care for baby born to an HIV positive mother (e.g., administration of ARV and appropriate feeding)		
F19	I can document newborn diagnosis and care in appropriate registers		
F20	I provide appropriate postpartum family planning care		
F21	I can insert postpartum IUCD		
F22	I can provide timely immunization services to newborns and infants according to the national guidelines		
F23	I can educate women and families for timely and complete immunization of newborns and infants based on national guidelines		

7. **Midwives provide a range of individualized, culturally sensitive abortion-related care services for women requiring or experiencing pregnancy termination or loss that are congruent with applicable laws and regulations and in accord with national protocols**

a. Based on my own assessment of my knowledge, **in general** I rate **my knowledge** of policies, theories and principles to provide **a range of individualized, culturally sensitive abortion-related care services for women needing or going through termination of pregnancy/miscarriage according to the national guidelines** to be as:

(Move the slider to rate your self-assessment of knowledge in this area on a scale of 0 to 100 OR place a cross on the line below to mark your assessment of self-knowledge on a scale of 0-100 WHERE the left end point of line denotes 0 knowledge and the right endpoint of the line denotes 100% knowledge)

b. **Do** regulations allow you to provide abortion services? Yes ☐ No ☐

c. If yes, which methods of abortion services can you provide independently? (tick all that apply)

d. Medical ab**ortion** ☐ **Vacuum aspiration** ☐ **Any other (please specify)** ________________________

**Item No. G**	**Skill Statements:**<br><br>**Midwives provide a range of individualized, culturally sensitive abortion-related care services for women requiring or experiencing pregnancy termination or loss that are congruent with applicable laws and regulations and in accord with national protocols**	**Not aware of this skill/not relevant**<br><br>**(Please tick√)**	**How confident are you to perform this skill independently?**<br>**1: Not confident**<br>**2: Little confident**<br>**3: Somewhat confident**<br>**4: Quite confident**<br>**5: Very Confident**<br>**(Please mark your self-rating)**
G01	I can assess the gestational age in a woman requiring abortion services		
	**I can counsel the women seeking abortion services on**		
G02	- Choice to abort		
G03	- Choice to continue pregnancy		
G04	- Methods of obtaining abortion		
G05	- Post abortion sexual activity and family planning		
G06	I can collect comprehensive medical history to identify contraindications to a method of abortion (medical, aspiration)		
	**In case of abortion, I can assess for:**		
G07	- Involution of uterus		
G08	- Self-care (including hygiene)		
G09	- Abortion related complication (sepsis, perforation)		
G10	- I can prescribe and/or dispense contraceptives to the couple		
G11	I can perform manual aspiration of the uterus upto 12 completed weeks of pregnancy		

Barriers in the practice of midwifery				
		Do you agree to this?		If yes, to what degree does this affect your ability to provide quality midwifery services?<br><br>1= No effect<br>**2**= Little effect<br>**3**= Some effect<br>4= Quite a lot of effect<br>5= Great effect
		Yes	No	
**Socio-Cultural**	**Beliefs:**<br>1. I believe society sees the midwife is seen as "dirty" and "polluted".			
	2. I believe midwifery status as a profession is low.			
	3. I believe midwifery does not have a strong professional identity.			
	4. I believe powerful posts (higher level) who make decisions which affect midwives are usually occupied by men.			
**Economic**	**Beliefs**:<br>1. I have a low salary that is difficult to live on.			
	3. There is a lack of governmental financial commitment to midwifery.			
	4. I believe my hostel facilities at school were not healthy.			
	5. I am required to do excessive overtime.			
	6. I believe there is a lack of safety and security when I am working.			
**Professional**	**Beliefs:**<br>1. I believe where I work there are inadequate resources (lack of supplies, water, sanitation, medications and/or equipment).			
	2. I believe midwifery is often confused with nursing.			
	3. I believe there is a lack of consistent use of standards and guidelines at work			
	4. I believe where I work there are inadequate resources (lack of supplies, water, sanitation, medications and/or equipment).			
	5. I believe there are restrictions on my ability to practice midwifery.			
	6. I believe I had inadequate training that impacted my confidence.			
	7. I believe my mentors lacked clinical experience.			
