# Supplementary material for: Developing and validating a self-assessment tool for assessing confidence of nurse-midwives against competency domains of the International Confederation of Midwives, in India
Source: PLOS Glob Public Health. 2024 Oct 23;4(10):e0003766. doi: 10.1371/journal.pgph.0003766 (PMC11498683; doi:10.1371/journal.pgph.0003766)
Supplement: Attachment — Submitted filename: Comments on PGPH.docx [file pgph.0003766.s004.docx]

**Comments on PGPH-D-23-02310**

Developing and validating a self-assessment tool for assessing confidence of Indian

Nurse-Midwives on competency domains of the International Confederation of

Midwives

**General**

This is a useful study which should be publishable following some minor amendments and clarifications.

**Title long version**

Suggest editing to: Developing and validating a self-assessment tool for assessing confidence of Nurse-Midwives on competency domains of the International Confederation of Midwives in India

**Abstract**

Line 30-37

This looks like a clear summary. I suggest adding a brief explainer /definition of nurse-midwife. Those roles should not be conflated, the abstract starts with reference to midwives and midwifery care and midwifery cadre, then jumps to nurse-midwife. E.g transition to professional midwifery from ?

Line 43

There is no evidence of transferability to high-income countries, please provide further explanations on how this can be applied to high-income when setting is low-middle income countries.

**Background**

Line 80 onwards

Please provide information on educational pathways /degree level of the nursing programme too, as there is no context of the add-on 18 months. Are those programmes provided by higher education institutions- please add brief contextual explainer.

Line 130

Please expand on the concept of ‘situated competence’.

**Methodology**

Line 138

Please clarify why chosen for mortality ratio, it is unclear.

Line 151

Do labour rooms refer to birth rooms? Or nurse-midwife only provide labour care. Please clarify: expand or edit.

Line 158

Please remove first author reference, irrelevant here. Obvious from author list who is the 1^st^ author. Please describe iterations instead and how team members worked together, removed/minimised bias and agree or found resolution when in disagreement.

Line 161

Please remove *African countries*: clarify which countries instead

Line 162

Again the reference of first author having authored other papers is completely irrelevant for the purpose of the paper, remove.

Line 177

Refer to nurse-midwives in India and not ‘indian’

**Results**

**Please report absolute numbers throughout not just percentages. E.g 97% females add (n. x, 97%).**

Line 229

Please expand on participants age. It is unclear how they can be qualified nurse-midwife at 18 years old.

Line 231

Please give context to employment/employer status (e.g public health facilities)

Table 2 and Line 235

Please clarify timeframe of birth attendance (how many births in how long/throughout their career or last 2 weeks as above).

Discussion

Please amend the discussion to include reference to existing literature, what is new and how it compares with published literature.

Line 308

Please expand comparison to other existing tools
