# Supplementary material for: Developing and validating a self-assessment tool for assessing confidence of nurse-midwives against competency domains of the International Confederation of Midwives, in India
Source: PLOS Glob Public Health. 2024 Oct 23;4(10):e0003766. doi: 10.1371/journal.pgph.0003766 (PMC11498683; doi:10.1371/journal.pgph.0003766)
Supplement: Attachment — Submitted filename: Answer to reviewer comments.docx [file pgph.0003766.s005.docx]

| **Developing and validating a self-assessment tool for assessing confidence of Nurse-Midwives against competency domains of the International Confederation of Midwives, in India** | |
| --- | --- |
| **Reviewer 1.** |  |
| I thought that the technical content of the paper, around the description of the tool and its validation – the factor analysis etc. – was very strong. I didn’t have any technical comments on it. | **Thankyou** |
| My understanding is that you are arguing that: - We want to measure competence, but this is expensive/hard to do at scale/hard to do often through traditional measures - Instead, we can measure self-assessed confidence – that this is a good ‘proxy’ for competence and it is feasible to do this cheaply and at scale - This has been done previously for a subset of the ICM domains; the contribution of this tool is that it covers all ICM domains (I may have misunderstood this) - The findings show that it works as a tool, and can be contextualized - The contribution is therefore to offer a contextualizable tool for large scale, cost-effective assessments of confidence that proxy for competence This would be a clear justification (if I have understood it correctly). However, I don’t feel you set this out clearly in the beginning. | **Thankyou. You are right. We have revised the text now** |
| The background second para (line 64 onwards) doesn’t convincingly make a case as to why we cannot rely on existing approaches to measure competence for certain needs (e.g., large scale assessments) – which you do well starting line 283 but that is far too late. I would have expected you to set out clearly what the problem is, that this article is seeking to solve. Without this, it is not clear why you start talking about confidence when the background is all about attempts to measure competence. A clear sentence or para that says: this is what the article does, and this is what it contributes, would be very helpful. | **We have revised the text and hope it reads better now.** |
| I would considering keeping the section on competence vs confidence to follow on directly from this section (after line 79), and then bring the Indian context after. At the moment, the Indian context section is in the middle of the two sections that provide the conceptual justification, which breaks the flow. | **Done** |
| On the competence vs confidence section, this would have a clearer narrative flow if you had established that we cannot measure competence easily at scale using traditional measures, so we need to measure confidence instead. I found this section a little weak – I would have expected a stronger theoretical and empirical justification as to why measuring confidence is a good proxy for measuring competence. I would have brought in some examples of where it has been successfully used elsewhere here. | **We have done this now** |
| On the methodology, I got a little bit confused about this sentence: “At the second stage, nurse-midwives were randomly selected from the clinical settings linked to each selected education institution” (line 145). Do you mean you got a list of students who had graduated from the selected teaching institutions, and went and found them wherever they were working? Or they are only from clinical settings attached to the education institution, and not other facilities across the states? In which case how representative are they? This needs more clarification and explanation. | Yes we selected participants only from clinical settings attached to the education institution, and not other facilities across the states. We have mentioned this and described it in the limitations |
| On the evaluating section (line 210), it would be helpful if you set out: to test the validity of the tool, we need to test whether it has good internal consistency, reliability etc. etc. And to do this, we do (e.g., factor analysis). You jump straight into the weeds of how you do these tests without setting out why these are the right tests to demonstrate that the tool works. For a non-technical reader, some clearer framing would be helpful. | **We have modified the text** |
| In the results section – line 235 – is the 75% figure right? | **We had** added the percentage of “30% (671/2198) having assisted up to six births + 47% (1026/2198) having assisted more than seven births in the last two weeks of survey. The total comes to 77% nurse-midwives who were practicing at the time of the survey. 75% is a typing mistake. |
| I think if you set up the purpose of the article more clearly, you would also be able to reshape the discussion into a clearer narrative. And the abstract also. | **Have tried to do this** |
| I did wonder whether you wanted to present some of the findings of the tool to justify its utility (not just its validity). For example, you say that ‘another strength of this validated tool is that it enables a quick and efficient way to identify skill gaps’ – do you have some examples of this? What did the findings enable in terms of policy or programmatic change? | Though usually a paper describing the validation process does not present results. Results are presented as a separate paper. However, we have added a few lines as an example from your suggestion |
| For the external validity point, perhaps you could reflect on the process you followed for India (content and face validity) and what it would take to tailor this tool to a different context? | Added some lines in the discussion. The tool has been successfully adapted in Bangladesh since it was used in India |
|  |  |
| **Reviewer 2** |  |
| Suggest editing to: Developing and validating a self-assessment tool for assessing confidence of Nurse-Midwives on competency domains of the International Confederation of Midwives in India | Accepted |
| **Abstract**  Line 30-37  This looks like a clear summary. I suggest adding a brief explainer /definition of nurse-midwife. Those roles should not be conflated, the abstract starts with reference to midwives and midwifery care and midwifery cadre, then jumps to nurse-midwife. E.g transition to professional midwifery from ? | We have modified as per suggestion |
| Line 43  There is no evidence of transferability to high-income countries, please provide further explanations on how this can be applied to high-income when setting is low-middle income countries. | We have removed the word high |
| **Background**  Line 80 onwards  Please provide information on educational pathways /degree level of the nursing programme too, as there is no context of the add-on 18 months. Are those programmes provided by higher education institutions- please add brief contextual explainer. | Though we had some description, we have tried to improve upon it |
| Line 130  Please expand on the concept of ‘situated competence’. | Done |
| **Methodology**  Line 138  Please clarify why chosen for mortality ratio, it is unclear. | Knowing that nurse-midwives contribute to quality maternal and newborn services, we assumed they would have an impact on maternal mortality. Therefore, the six states were selected on the basis of their Maternal Mortality Ratios (MMR) of the time the study was conceptualised |
| Line 151  Do labour rooms refer to birth rooms? Or nurse-midwife only provide labour care. Please clarify: expand or edit. | Labour rooms are where childbirth occurs. There are no birth rooms in India (unless birth rooms are also labour rooms?).  As described from lines 142 to 152, nurse-midwives are posted in antenatal, labour rooms and postnatal wards, but they are not the same nurse-midwives. A set of midwives only perform antenatal, or postnatal tasks, and another set assists labour in labour rooms. We have revised the text to be clearer. |
| Line 158  Please remove first author reference, irrelevant here. Obvious from author list who is the 1^st^ author. Please describe iterations instead and how team members worked together, removed/minimised bias and agree or found resolution when in disagreement. | Done. Since the skill statements were from the ICM, these were found appropriate by researchers involved in these various iterations. However, each statement was revised in terms of the local words used within each country. |
| Line 161  Please remove *African countries*: clarify which countries instead | **Done** |
| Line 162  Again the reference of first author having authored other papers is completely irrelevant for the purpose of the paper, remove. | **Done** |
| Line 177  Refer to nurse-midwives in India and not ‘indian’ | **Done** |
| **Results**  **Please report absolute numbers throughout not just percentages. E.g 97% females add (n. x, 97%).** | **Done** |
| Line 229  Please expand on participants age. It is unclear how they can be qualified nurse-midwife at 18 years old. | Sorry for typo mistake. We have corrected the age range after verifying. |
| Line 231  Please give context to employment/employer status (e.g public health facilities) | Public health facilities here means all government owned and run hospitals across all states opposed to private hospitals |
| Table 2 and Line 235  Please clarify timeframe of birth attendance (how many births in how long/throughout their career or last 2 weeks as above). | **Revised the text as follows;**  While 3% of the participants (72/2198) had no clinical experience, 9% (202/2198) had no clinical experience in midwifery services. As seen in table-2, almost 77% of the participants were in practice of assisting births as measured by “births assisted in preceding two weeks of survey”; 30% (671/2198) having assisted up to six births and 47% (1026/2198) having assisted more than seven births in the last two weeks of survey. |
| Discussion  Please amend the discussion to include reference to existing literature, what is new and how it compares with published literature. | Done but in the interest of word limit we have shifted some references from the introduction to the discussion |
| Line 308  Please expand comparison to other existing tools |  |
